# Large variations in the practice patterns of surgical antiseptic preparation solutions in patients with open and closed extremity fractures: a cross-sectional survey

**DOI:** 10.1186/s13756-018-0440-z

**Published:** 2018-11-29

**Authors:** Maria Jurado-Ruiz, Gerard P. Slobogean, Sofia Bzovsky, Alisha Garibaldi, Nathan N. O’Hara, Andrea Howe, Brad Petrisor, Sheila Sprague

**Affiliations:** 1 Department of Trauma and Orthopaedic Surgery, Hospital Universitari Vall d’Hebron, Universitat Autonoma de Barcelona, Barcelona, Spain; 2 R Adams Cowley Shock Trauma Center, Department of Orthopaedics, University of Maryland School of Medicine, Baltimore, MD USA; 3 Division of Orthopaedic Surgery, Department of Surgery, McMaster University, Hamilton, ON Canada; 4 Department of Health Research Methods, Evidence, and Impact, McMaster University, Hamilton, ON Canada; 5 McMaster University, 293 Wellington Street North, Suite 110, Ontario, Hamilton L8L 8E7 Canada

**Keywords:** Survey, Open fracture, Closed fracture, Antiseptic preparation, Surgical site infection, Surgical preparation, Antiseptic solution

## Abstract

**Background:**

Surgically-managed fractures, particularly open fractures, are associated with high rates of surgical site infections (SSIs). To reduce the risk of an SSI, orthopaedic surgeons routinely clean open fracture wounds in the emergency department (ED) and then apply a bandage to the open wound. Prior to the surgical incision, it is standard practice to prepare the fracture region with an antiseptic skin solution as an additional SSI prevention strategy. Multiple antiseptic solutions are available.

**Objectives:**

To explore the variation in practice patterns among orthopaedic surgeons regarding antiseptic solution use in the ED and antiseptic preparatory techniques for fracture surgery.

**Methods:**

We developed a 27-item survey and surveyed members of several orthopaedic associations.

**Results:**

Two hundred and-ten surveys were completed. 71.0% of respondents irrigate the open wound and skin in the ED, primarily with saline alone (59.7%) or iodine-based solutions (32.9%). 90.5% of responders indicated that they dress the open wound in the ED, with 41.0% applying a saline-soaked bandage and 33.7% applying an iodine-soaked dressing (33.7%). In their surgical preparation of open fractures, 41.0% of respondents used an iodine-based solution, 26.7% used a chlorhexidine gluconate (CHG)-based solution, and 31.4% used a combination of the two. In closed fractures, 43.8% of respondents used a CHG-based solution, 28.1% used an iodine-based solution, and 27.1% used a combination. Despite theoretical concerns about the use of alcohol in open wounds, 51.4% used alcohol-based solutions or alcohol alone during skin preparation of open fractures.

**Conclusions:**

A lack of consensus exists regarding use of antiseptic surgical preparation solutions for fractures. High-quality clinical research is needed to assess the effectiveness of different surgical antiseptic preparation solutions on patient outcomes in fracture populations.

**Electronic supplementary material:**

The online version of this article (10.1186/s13756-018-0440-z) contains supplementary material, which is available to authorized users.

## Introduction

Surgically managed fractures have a high incidence of surgical site infections (SSIs) as compared to other surgical specialties [1]. SSIs can be devastating to patients and to their families, as they may lead to use of additional antibiotic use, additional surgical interventions, prolonged morbidity, loss of function, potential limb loss, and even death [2]. Given the negative consequences of an SSI, the prevention of infection is an important focus of perioperative fracture management.

To reduce the risk of an SSI in open fracture management, orthopaedic surgeons often irrigate the open fracture wound in the emergency department and then apply a bandage, either dry or soaked with saline or an antiseptic solution. Additionally, prior to a surgical incision for operative management of either open or closed fractures, it is standard practice to prepare the injured region with an antiseptic solution to reduce the risk of an SSI.

In general, the application of an antiseptic solution prior to surgical incision is known to be effective in preventing SSIs [3]. However, given the many types of antiseptic solutions with different active agents available to surgeons, there is no clear evidence on the best antiseptic solution for fracture patients [3]. Two common preoperative skin antiseptics used are chlorhexidine gluconate (CHG) and iodine-based solutions, both of which have a strong biological rationale [4] and are available in either an aqueous-based or alcohol-based solution.

There is a paucity of compelling clinical evidence regarding which of these surgical antiseptic preparation solutions is more effective at preventing SSIs [3] and the evidence guiding preoperative antiseptic skin solution choice in fracture surgery (either open or closed) is largely extrapolated from other surgical disciplines [3]. Data from the FLOW trial (Fluid Lavage of Open Wounds) of approximately 2500 open fracture patients found great variation in the early management of open fractures and the type of preoperative skin preparations used by surgeons [5].

To quantify this further, we conducted a survey of orthopaedic surgeons to understand the practice patterns regarding use of antiseptic solutions in the emergency department and surgical antiseptic preparatory processes for open and closed extremity fractures.

## Materials and methods

### Questionnaire development - item generation

We developed a questionnaire using the previous literature and key informants to examine preferences and practices of orthopaedic surgeons for early management of open fracture wounds, and the preoperative antiseptic skin preparation practices for open and closed extremity fractures. The survey was reviewed by five experts, including three orthopaedic surgeons and two epidemiologists, to ensure that nothing vital was missed and to ensure that the wording of the questions was clear and precise.

### Pretesting and validity assessments of the questionnaire

We pretested the questionnaire in an independent group of five orthopaedic surgeons with experience in clinical research and the treatment of open and closed fractures to evaluate whether the questionnaire adequately encompassed current management practices (face validity), and whether the individual questions adequately addressed the objectives of the current study (content validity). This group of surgeons also commented on the clarity and comprehensiveness of the questionnaire. The survey was revised based upon the recommendations of this independent group and retested in the same group until no additional issues or concerns were identified.

### Survey description

The final survey was comprised of 27 questions using check-boxes and brief open-ended questions. All of the questions used clear and widely recognized terminology to enhance the validity of results. The survey length was kept to a minimum to maximize response rate and to limit respondent fatigue. The survey included questions about the participants’ demographics, location of practice, type of practice (community or academic), and their number of years of experience treating fracture patients. It also included questions about early open fracture wound management, the use of single or multiple surgical antiseptics for both open and closed fractures, type(s) of antiseptics used, irrigation procedures, the use of dressings on open wounds, and what typically guides surgeons’ clinical decision making, as well as their perceptions on the importance of antiseptic selection in the prevention of an SSI. Finally, respondents were asked several questions about their personal interest in participating in future clinical trials comparing different surgical antiseptic preparation solutions. All questions were in the context of the preoperative surgical care of fracture patients with the focus being on how to prevent SSI of the current fracture rather than the prevention of secondary infections. The questions were not randomized and we used adaptive questioning for certain items. All questions fit an average screen, with 1 question per page. All questions had to be completed, and respondents were able to go back and review their previous answers. Please refer to Additional file 1 for a copy of the survey.

### Survey administration

The survey was made available to 2149 active members of the Canadian Orthopaedic Association (COA), Canadian Orthopaedic Trauma Society (COTS), and the Orthopaedic Trauma Association (OTA) through email messages from the corresponding association. The e-mail included a link to the online survey and information about the survey’s purpose, how their data would be stored and used, the length of time required to complete the survey, and the investigators’ contact information. Follow-up emails at 2 weeks and at 4 weeks following the initial request were sent to aid in increasing the response rate among survey participants. Participants were assigned a unique ID when opening the link to our survey which they would only be able to answer once; the IP address of each client computer was also used to identify any potential duplicate entries from the same user. Completion of the survey was considered as implicit consent. All surveys were completed using SurveyMonkey, an online survey development cloud-based software that includes data analysis, sample selection, bias elimination, and data representation tools. No monetary incentives or pre-notification telephone calls were used for this survey. Questionnaire completion was voluntary and individual responses were kept confidential, only being accessible through a password protected account. The data was collected from November 2016 to May 2017.

### Sample size

To determine the number of respondents needed to sufficiently power our analysis, we assumed that approximately 50% of surgeons surveyed would use a surgical antiseptic preparation solution with chlorhexidine gluconate and 50% of surgeons would use a surgical antiseptic preparation solution with iodine as their preferred choice. The calculation for appropriate sample size was performed according to the following formula, assuming a 95% confidence interval for the estimate: *N* = (Z_α_/w)^2^ p(1 - p), [Where: Z = z value (1.96 for 95% confidence interval); w = width of the confidence interval, expressed as decimal (0.07 to give ±7); *p* = hypothesized proportion of who would use a surgical antiseptic preparation solution with chlorhexidine gluconate, expressed in decimal (70% = 0.70)]. According to our calculation, approximately 164 completed questionnaires would facilitate a meaningful analysis.

### Statistical analysis

We summarized all categorical and dichotomous variables with frequencies and percentages. Only completed questionnaires were analyzed. Continuous data were described with means and standard deviations (SDs). All analyses were conducted in SPSS Version 25.0 (IBM Corporation, Armonk, NY).

## Results

### Characteristics of the respondents

A total of 210 surveys were completed. Most of the respondents were from North America (45.2%), Europe (25.7%), or Asia (18.1%) (Table 1). Nearly 93% of the respondents were men and the mean age was 47.7 ± 8.2 years. Approximately three-quarters of the respondents practiced in an academic teaching hospital and most respondents (82.4%) had 10 or more years’ experience treating fracture patients.

**Table 1:** Characteristics of survey respondents

Characteristic	Number of respondents (*N* = 210), n (%)
Location	
North America	95 (45.2)
Europe	54 (25.7)
Asia	38 (18.1)
Australasia	7 (3.3)
Africa	8 (3.8)
Central and South America	8 (3.8)
Sex	
Male	195 (92.9)
Female	15 (7.1)
Mean age ± SD (years)	47.7 ± 8.2
Location of practice	
Academic	153 (72.9)
Community	57 (27.1)
Number of years treating fracture patients	
< 10 years	37 (17.6)
≥ 10 years	173 (82.4)

### Solutions used in the emergency room to treat open fracture wounds

More than two-thirds (149/210 = 71.0%) of the responders indicated that they irrigate the open wound and skin in the emergency department. Among these 149 surgeons, almost two-thirds, indicated that the irrigation is conducted following orthopaedic surgeon consultation (95/149 = 63.8%), whereas a third indicated that the irrigation is performed immediately upon arrival to emergency department (52/149 = 34.9%). Among those who irrigated the open wound in the emergency room, more than half (89/149 = 59.7%) used saline alone, 32.9% used an aqueous-iodophor solution (e.g. Betadine®), and 7.4% used either CHG or iodine in an alcohol-based solution (Table 2).

**Table 2:** Agents used among surgeons who irrigate the open fracture wound and skin in the emergency department

Agent	Number of Respondents (*N* = 149*), n (%)
Saline	89 (59.7)
Iodine Aqueous-Based Solution	37 (24.8)
CHG Alcohol-Based Solution	7 (4.7)
Hydrogen Peroxide	6 (4.0)
Aqueous-Based CHG Solution	5 (3.4)
Iodine Alcohol-Based Solutions	4 (2.7)
Soap	1 (0.7)

^*^149/210 surgeons irrigate in the emergency department

Most surgeons indicated that they always dress the open wound in the emergency department (90.5%) and approximately one-third used a sterile dry-dressing (75/205 = 36.6%). 41.0% (84/205) used a saline-soaked dressing and a third indicated that they use an iodine-soaked dressing (69/205 = 33.7%). Please note that surgeons were able to select more than one response when indicating the type of dressing used.

### Open fractures – Surgical preparation practices

Surgeons were asked if it was in their routine practice to use only a single antiseptic surgical preparation solution or multiple antiseptic surgical preparation solutions when preparing an open fracture for surgery. 53.8% (113/210) of the respondents indicated that they routinely use multiple antiseptic surgical preparation solutions when preparing an open fracture for surgery (Table 3). Surgeons were then asked to indicate from the following, 1) CHG in an alcohol-based solution, 2) CHG in an aqueous-based solution, 3) aqueous-iodophor solution, 4) alcohol-iodophor solution, and 5) alcohol, what type of antiseptic surgical preparation solution(s) they used when preparing an open fracture for surgery. For this question, surgeons were able to select more than one antiseptic surgical preparation solution as a response. 41.0% of the respondents indicated that they use only iodine-based solution(s) (either aqueous- or alcohol-based) in their preparation practices for open fractures, 26.7% indicated the use of only CHG-based solution(s) (either aqueous- or alcohol-based), and 31.4% indicated that they use a combination of both CHG-based and iodine-based solutions (either aqueous- or alcohol-based) (Table 4). 51.4% of the respondents indicated that they used an alcohol-based solution or added alcohol alone in their preparation practices for open fractures. Specifically, 35.7% used an alcohol-CHG preparation solution (Chloraprep®, Soluprep® or equivalents), 7.6% (16/210) used an alcohol-iodophor preparation solution (Duraprep™ and equivalents), and 20.0% added alcohol alone as part of the surgical preparation routine. While the results of this survey suggest that many orthopaedic surgeons use alcohol-containing antiseptics or add alcohol alone for surgical skin preparation of open fractures, the orthopaedic community also indicated in the open-ended questions of the survey that it follows standard precautions regarding the use of alcohol antiseptic solutions in order to prevent pooling of the solutions and ensure that adequate drying (evaporation) takes place to avoid the flammability risks from electrocautery. Of total, we found 20 different preoperative antiseptic regimens in open fractures for preventing SSI in open fractures.

**Table 3:** Use of single or multiple surgical antiseptics in open and closed fractures

	Single Solution		Multiple Solutions^a^		Total^a^	
Type of Solution	Open Fractures (*N* = 97) n (%)	Closed Fractures (*N* = 72) n (%)	Open Fractures (*N* = 113) n (%)	Closed Fractures (*N* = 138) n (%)	Open Fractures (*N* = 210) n (%)	Closed Fractures (*N* = 210) n (%)
Aqueous-Iodophor Solution (Betadine®, ScrubCare® and equivalents)	66 (68.0)	29 (40.3)	79 (69.9)	73 (52.9)	145 (69.0)	102 (48.6)
Aqueous-CHG Solution (Betasept®, Hibiclens® or equivalents)	11 (11.3)	2 (2.8)	69 (61.1)	89 (64.5)	80 (38.1)	91 (43.3)
Alcohol-Iodophor Solution (Duraprep™ and equivalents)	2 (1.0)	8 (11.1)	14 (12.4)	21 (10.0)	16 (7.6)	29 (13.8)
Alcohol-CHG Solution (Chloraprep®, Soluprep® or equivalents)	16 (7.6)	31 (43.1)	59 (52.2)	90 (42.9)	75 (35.7)	121 (57.6)
Alcohol	2 (1.0)	2 (2.8)	40 (35.4)	51 (24.3)	42 (20.0)	53 (25.2)

^a^Numbers may not add up to N value due to ability to select multiple responses

**Table 4:** Breakdown of types of surgical antiseptics used in open and closed fractures

Antiseptic surgical preparation solution	Open Fractures *N* = 210 n (%)	Closed Fractures *N* = 210 n (%)	Total *N* = 420 n (%)
Use of CHG-Based Solutions Only	56 (26.7)	92 (43.8)	148 (35.2)
CHG in an Aqueous-Based Solution + CHG in an Alcohol-Based Solution	20 (9.5)	40 (19.0)	60 (14.3)
CHG in an Alcohol-Based Solution	16 (7.6)	31 (14.8)	47 (11.2)
CHG in an Aqueous-Based Solution	11 (5.2)	2 (1.0)	13 (3.1)
CHG in an Aqueous-Based Solution + CHG in an Alcohol-Based Solution + Alcohol	6 (2.9)	10 (4.8)	16 (3.8)
CHG in an Alcohol-Based Solution + Alcohol	3 (1.4)	6 (2.9)	9 (2.1)
CHG in an Aqueous-Based Solution + Alcohol	0 (0.0)	3 (1.4)	3 (0.7)
Use of Iodine-Based Solutions Only	86 (41.0)	59 (28.1)	145 (34.5)
Only Aqueous-Iodophor Solution	66 (31.4)	29 (13.8)	95 (22.6)
Aqueous-Iodophor Solution + Alcohol	11 (5.2)	14 (6.7)	25 (6.0)
Aqueous-Iodophor Solution + Alcohol-Iodophor Solution	4 (1.9)	5 (2.4)	9 (2.1)
Aqueous-Iodophor Solution + Alcohol-Iodophor Solution + Alcohol	3 (1.4)	3 (1.4)	6 (1.4)
Only Alcohol-Iodophor Solution	2 (1.0)	8 (3.8)	10 (2.4)
Combined Use of CHG-Based and Iodine-Based Solutions	66 (31.4)	57 (27.1)	123 (29.3)
CHG in an Aqueous-Based Solution + Aqueous- Iodophor Solution	25 (11.9)	10 (4.8)	35 (8.3)
CHG in an Alcohol-Based Solution + Aqueous- Iodophor Solution	14 (6.7)	13 (6.2)	27 (6.4)
CHG in an Aqueous-Based Solution + Aqueous- Iodophor Solution + Alcohol	9 (4.3)	8 (3.8)	17 (4.0)
CHG in an Alcohol-Based Solution + Aqueous- Iodophor Solution + Alcohol	4 (1.9)	2 (1.0)	6 (1.4)
CHG in an Aqueous-Based Solution + CHG in an Alcohol-Based Solution + Aqueous-Iodophor Solution	3 (1.4)	9 (4.3)	12 (2.9)
CHG in an Alcohol-Based Solution + Alcohol-Iodophor Solution	3 (1.4)	2 (1.0)	5 (1.2)
CHG in an Alcohol-Based Solution + Aqueous-Iodophor Solution + Alcohol-Iodophor Solution	2 (1.0)	3 (1.4)	5 (1.2)
CHG in an Aqueous-Based Solution + Alcohol- Iodophor Solution	2 (1.0)	3 (1.4)	5 (1.2)
CHG in an Alcohol-Based Solution + Alcohol-Iodophor Solution + Alcohol	0 (0.0)	1 (0.5)	1 (0.2)
CHG in an Aqueous-Based Solution + CHG in an Alcohol-Based Solution + Aqueous-Iodophor Solution + Alcohol	4 (1.9)	2 (1.0)	6 (1.4)
CHG in an Aqueous-Based Solution + CHG in an Alcohol-Based Solution + Aqueous-Iodophor Solution + Alcohol-Iodophor Solution	0 (0.0)	1 (0.5)	1 (0.2)
CHG in an Aqueous-Based Solution + Aqueous- Iodophor Solution + Alcohol-Iodophor Solution	0 (0.0)	1 (0.5)	1 (0.2)
CHG Aqueous-Based Solution + Aqueous-Iodophor Solution + Alcohol-Iodophor Solution + Alcohol	0 (0.0)	1 (0.5)	1 (0.2)
CHG in an Aqueous-Based Solution + CHG in an Alcohol-Based Solution + Aqueous-Iodophor Solution + Alcohol-Iodophor Solution + Alcohol	0 (0.0)	1 (0.5)	1 (0.2)
Use of Alcohol Only as a Single Solution	2 (1.0)	2 (1.0)	4 (1.0)

### Closed fractures – Surgical preparation practices

Surgeons were asked if it was in their routine practice to use only a single antiseptic surgical preparation solution or multiple antiseptic surgical preparation solutions when preparing a closed fracture for surgery. 65.7% (138/210) of the respondents indicated that they routinely use multiple antiseptic surgical preparation solutions when preparing a closed fracture for surgery (Table 3). Surgeons were then asked to indicate from the following, 1) CHG in an alcohol-based solution, 2) CHG in an aqueous-based solution, 3) aqueous-iodophor solution, 4) alcohol-iodophor solution, and 5) alcohol, what type of antiseptic surgical preparation solution(s) they used when preparing a closed fracture for surgery. For this question, surgeons were able to select more than one antiseptic surgical preparation solution as a response. 43.8% of the respondents indicated that they use only CHG-based solution(s) (either aqueous- or alcohol-based) in their preparation practices for closed fractures, 28.1% indicated the use of only iodine-based solution(s) (either aqueous- or alcohol-based), and 27.1% of respondents indicated that they use a combination of both CHG-based and iodine-based solutions (either aqueous- or alcohol-based) (Table 4). Most of the respondents used an alcohol-based solution or alcohol alone (169/210 = 80.5%) in their preparation practices for closed fractures. Specifically, 57.6% (121/210) used an alcohol-CHG preparation (Chloraprep®, Soluprep® or equivalents), 25.2% (53/210) added alcohol alone, and 13.8% (29/210) used an alcohol-iodophor preparation (Duraprep™ and equivalents) solution as part of the preparation practices. Of total, we found 26 different preoperative antiseptic regimens in closed fractures for preventing SSI in closed fractures.

### Factors influencing surgeons’ decision to use surgical antiseptic solution(s)

Surgeons were able to select more than one response when indicating what factors influence their decision to use surgical antiseptic preparation solution(s) when preparing a fracture for surgery. Surgeons indicated that their decision to use antiseptic surgical preparation solution(s) in open fractures was influenced by hospital and/or operating room policy (46.7%), personal experience (43.3%), and experience during training (38.1%). Choice of antiseptic surgical preparation solution(s) in closed fractures was influenced by hospital and/or operating room policy (57.6%), literature (41.9%), and personal experience (39.0%) (Table 5).

**Table 5:** Factors influencing surgeons’ decisions to use surgical antiseptic solution(s), consideration in the election of the proper antiseptic, and importance to reduce risk of infection

	Open Fractures (*N* = 210) n (%)	Closed Fractures (*N* = 210) n (%)
Factors influencing surgeons’ decision^a^		
Hospital policy	98 (46.7)	121 (57.6)
Literature	77 (36.7)	88 (41.9)
Clinical experience	91 (43.3)	82 (39.0)
Practice guidelines	75 (35.7)	67 (31.9)
Experience during training	80 (38.1)	59 (28.1)
Colleague’s recommendation	31 (14.8)	20 (9.5)
Suppliers agreement	4 (1.9)	5 (2.4)
How often surgeons consider what type of antiseptic solution to use for open fracture cases		
Never (0%)	31 (14.8)	43 (20.5)
Infrequently (1–25%)	16 (7.6)	20 (9.5)
Sometimes (25–75%)	14 (6.7)	7 (3.3)
Usually (75–99%)	17 (8.1)	19 (9.0)
Always (100%)	132 (62.9)	121 (57.6)
Importance of type of antiseptic used in reducing risk of infection		
Not important	4 (1.9)	8 (3.8)
Slightly important	23 (11.0)	25 (11.9)
Moderately important	49 (23.3)	56 (26.7)
Very important	71 (33.8)	64 (30.5)
Extremely important	63 (30.0)	57 (27.1)

^a^Numbers may not add up to N value due to ability to select multiple responses

### The need for future research

More than half of the respondents (54.3%) supported the idea of participating in a randomized controlled trial comparing different surgical antiseptic preparation solutions for both open and closed fractures. Almost half of the surgeons who completed the survey (45.2%) indicated that they would participate in a randomized controlled trial comparing different antiseptic preparation solutions in the emergency room setting.

## Discussion

The results of this survey demonstrate that, 1) there is no established consensus among surgeons regarding the use of antiseptic preparation agents for early open fracture wound management in the emergency room and 2) there is no consensus regarding the use of preoperative skin antiseptics for the prevention of SSIs in the operative treatment of both open and closed fractures. The majority of surveyed surgeons indicated that they routinely perform an irrigation of the open fracture wound in the emergency department; however, there is a lack of consensus on the type of solution used. While most surgeons concur that a dressing should be used; there is a clear divide in the type of dressing they use, as approximately one-third use a sterile dry dressing, one-third use a saline soaked dressing, and the final third use a dressing soaked in an iodophor aqueous-based solution.

Furthermore, we found 20 different preoperative antiseptic regimens in the operative management of open fractures and 26 different preoperative antiseptic routines for preventing SSIs in the operative management of closed fractures. In both open and closed fractures, there was a lack of consensus on the use of iodine-based solutions and CHG-based solutions. In closed fractures, the majority of respondents used an alcohol-based solution. In open fractures, many surgeons are using alcohol-based solutions in this setting (51.4%), with alcohol-based CHG solutions being the most commonly used alcohol-based solutions (35.7%).

Our survey results indicate that when deciding what particular surgical antiseptic solution to use surgeons are less influenced by evidence from the literature for open fractures than for closed fractures; this finding suggests that there is a lack of available clinical evidence on this matter. If present, this evidence could help guide orthopedic surgeons in making decisions regarding what the best antiseptic solutions are for open fractures.

Overall, the results of this study must be interpreted in the context of the study design. Few surgeons outside of North America and Europe completed our survey, limiting the generalizability of our results in other regions. Additionally, orthopaedic organizations distributed the survey to their membership as part of their mailers or posted the survey on their website. This passive approach likely contributed to a low response rate and possible respondent bias. Previous surveys, where the researchers were able to contact potential survey participants directly, have achieved higher response rates [6, 7], which increases generalizability and decreases the risk of bias. This study is strengthened by the use of a rigorous process for the development of the questionnaire and extensive piloting of the survey. Moreover, our survey used open-ended questions about the surgical antiseptic preparation process to allow the participants to describe their practice pattern in detail without being restricted to categorical responses.

## Conclusion

In conclusion, there is a paucity of compelling clinical evidence examining which of these surgical antiseptic preparation solutions is more effective at preventing SSIs; we believe this has led to considerable controversy and practice variation among surgeons. High-quality clinical research is needed to resolve this debate and determine the effectiveness of different surgical antiseptic preparation solutions on patient important outcomes in open and closed fracture patients. Approximately half of the respondents endorsed the idea of participating in a randomized controlled trial comparing different surgical antiseptic preparation solutions indicating that the orthopaedic surgery community is interested in definitively resolving this question.

## Additional file

Additional file 1: Survey questions on practice patterns of surgical antiseptic preparation solutions in patients with open and closed extremity fractures. (DOCX 118 kb)

## Abbreviations

CHG: Chlorhexidine Gluconate
COA: Canadian Orthopaedic Association
COTS: Canadian Orthopaedic Trauma Society
ED: Emergency Department
FLOW: Fluid lavage of open wounds
OTA: Orthopaedic Trauma Association
SDs: Standard deviations
SSI: Surgical site infection
