# Supplementary material for: Large variations in the practice patterns of surgical antiseptic preparation solutions in patients with open and closed extremity fractures: a cross-sectional survey
Source: Antimicrob Resist Infect Control. 2018 Nov 29;7:148. doi: 10.1186/s13756-018-0440-z (PMC6267892; doi:10.1186/s13756-018-0440-z)
Supplement: Supplementary file 1 — Survey questions on practice patterns of surgical antiseptic preparation solutions in patients with open and closed extremity fractures. (DOCX 118 kb) [file 13756_2018_440_MOESM1_ESM.docx]

**PREPARE Survey**

| Part 1: Demographic Questions   1. Where are you currently practicing? (Check one)   □ Canada □ USA □ Europe □ Asia □ South America □ Australia & New Zealand  □ Africa □ Other (Specify): ________________  2. What is your gender? (Check one)  □ Male □ Female  3. What is your age? years  4. Which of the following best describes the clinical site where you primarily practice? (Check one)  □ Academic hospital □ Community hospital  5. For how many years have you been treating open and closed fracture patients? (Check one)  □ Less than 10 years  □ Greater than or equal to 10 years  □ I do not treat fracture patients *(Thank you for your time. The remaining questions ask about fracture patient management, so please stop here.)  6. Approximately how many open and closed fractures are operated on annually at the clinical site where you primarily practice?  open fractures closed fractures  *******The following questions ask about your practice patterns regarding the surgical antiseptic prep process used for closed and open fractures. *******  Part 2: Closed Fractures   1. Please describe in detail the surgical antiseptic prep process that you typically use for closed fractures. *(For example, limb is cleaned using a BactoShield CHG (chlorhexidine gluconate) scrub brush, then rinsed with Betadine.)*        1. When preparing a closed fracture for surgery, is it your routine practice to use only a single antiseptic surgical prep solution or multiple antiseptic surgical prep solutions?   □ Single solution – select what type of antiseptic surgical prep solution is used (Check one):  □ Betadine (Povidone-iodine 7.5%)  □ Betasept (Chlorhexidine gluconate 4%)  □ Duraprep (Iodine povacrylex (0.7% available iodine) and isopropyl alcohol, 74% w/w)  □ Chloraprep (Chlorhexidine gluconate 2% w/v and isopropyl alcohol 70% v/v)  □ Soluprep (Chlorhexidine gluconate 2% and isopropyl alcohol 70%)  □ Alcohol  □ Other - specify:  □ Multiple solutions – select what types of antiseptic surgical prep solutions are used (Check all that apply):  □ Betadine (Povidone-iodine 7.5%)  □ Betasept (Chlorhexidine gluconate 4%s  □ Duraprep (Iodine povacrylex (0.7% available iodine) and isopropyl alcohol, 74% w/w)  □ Chloraprep (Chlorhexidine gluconate 2% w/v and isopropyl alcohol 70% v/v)  □ Soluprep (Chlorhexidine gluconate 2% and isopropyl alcohol 70%)  □ Alcohol  □ Other - specify:   1. Please provide any additional details about the surgical antiseptic prep solution(s) that you use for closed fractures:        1. What is your rationale for using your preferred antiseptic surgical prep solution(s) in patients with closed fractures?        1. What primarily guides your decision to use the above antiseptic surgical prep solution(s) in closed fractures (Check one)?   □ It is what I have always done  □ What is available at time (hospital / operating room policy)  □ What I used in training  □ Supplier agreement  □ Literature  □ Colleagues recommended  □ Practice guidelines  □ Other - specify:   1. In what proportion of closed fracture cases do you consider the type of antiseptic surgical prep solution(s) used? % 2. How important is the type(s) of antiseptic surgical prep solution(s) in reducing risk of infection in patients with closed fractures?   □ Not important  □ Slightly important  □ Moderately important  □ Very important  □ Extremely important   1. Please provide any comments or additional information:       Part 3: Open Fractures   1. Do you typically irrigate the open wound and skin in the emergency department?   □ Yes  If yes, select the agent(s) used (Check all that apply):  □ Betadine (Povidone-iodine 7.5%)  □ Betasept (Chlorhexidine gluconate 4%)  □ Duraprep (Iodine povacrylex (0.7% available iodine) and isopropyl alcohol, 74% w/w)  □ Chloraprep (Chlorhexidine gluconate 2% w/v and isopropyl alcohol 70% v/v)  □ Soluprep (Chlorhexidine gluconate 2% and isopropyl alcohol 70%)  □ Saline  □ Other - specify:  If yes, specify when irrigation is conducted:  □ Immediately upon arrival to emergency department  □ Following orthopaedic surgery consultation  □ Other – specify:  □ No     1. Do you routinely dress the open wound in the emergency department?   □ Yes, always – select what type of dressing is used (Check all that apply):  □ Dry dressing  □ Saline soaked dressing  □ Iodine soaked dressing  □ Chlorhexidine gluconate soaked dressing  □ Other - specify:  □ Combination - specify:  □ Yes, sometimes – select what type of dressing is used (Check all that apply):  □ Dry dressing  □ Saline soaked dressing  □ Iodine soaked dressing  □ Chlorhexidine gluconate soaked dressing  □ Other - specify:  □ Combination - specify:  □ No, never   1. Please describe in detail the surgical antiseptic prep process that you typically use for open fractures. *(For example, limb is cleaned using a BactoShield CHG (chlorhexidine gluconate) scrub brush, then rinsed with Betadine.)*        1. When preparing an open fracture for surgery, is it your routine practice to use only a single antiseptic surgical prep solution or multiple antiseptic surgical prep solutions?   □ Single solution – select what type of antiseptic surgical prep solution is used (Check one):  □ Betadine (Povidone-iodine 7.5%)  □ Betasept (Chlorhexidine gluconate 4%)  □ Duraprep (Iodine povacrylex (0.7% available iodine) and isopropyl alcohol, 74% w/w)  □ Chloraprep (Chlorhexidine gluconate 2% w/v and isopropyl alcohol 70% v/v)  □ Soluprep (Chlorhexidine gluconate 2% and isopropyl alcohol 70%)  □ Alcohol  □ Other - specify:  □ Multiple solutions – select what types of antiseptic surgical prep solutions are used (Check all that apply):  □ Betadine (Povidone-iodine 7.5%)  □ Betasept (Chlorhexidine gluconate 4%s  □ Duraprep (Iodine povacrylex (0.7% available iodine) and isopropyl alcohol, 74% w/w)  □ Chloraprep (Chlorhexidine gluconate 2% w/v and isopropyl alcohol 70% v/v)  □ Soluprep (Chlorhexidine gluconate 2% and isopropyl alcohol 70%)  □ Alcohol  □ Other - specify:   1. Please provide any additional details about the surgical antiseptic prep solution(s) that you use for open fractures:        1. What is your rationale for using your preferred antiseptic surgical prep solution(s) in patients with open fractures?        1. What primarily guides your decision to use the above antiseptic surgical prep solution(s) in open fractures (Check one)?   □ It is what I have always done  □ What is available at time (hospital / operating room policy)  □ What I used in training  □ Supplier agreement  □ Literature  □ Colleagues recommended  □ Practice guidelines  □ Other - specify:   1. In what proportion of open fracture cases do you consider the type of antiseptic surgical prep solution(s) used? % 2. How important is the type(s) of antiseptic surgical prep solution(s) in reducing risk of infection in patients with open fractures?   □ Not important  □ Slightly important  □ Moderately important  □ Very important  □ Extremely important   1. Please provide any comments or additional information:       Part 4: Trial Participation   1. Would you be willing to participate in a randomized controlled trial comparing different surgical antiseptic prep solutions?   □ Yes, for both open and closed fractures  □ Yes, for open fractures only  □ Yes, for closed fractures only  □ Unsure  □ No, not at this time   1. Would you be willing to participate in a randomized controlled trial comparing different surgical antiseptic prep solutions in the emergency room setting?   □ Yes  □ Unsure  □ No, not at this time   1. Please provide any comments or additional information:         **Thank you for your participation in this survey. The survey is now complete.** |
| --- |
